# Effect of extracorporeal shock wave therapy on the microbial community in burn scars: retrospective case–control study

**DOI:** 10.1097/JS9.0000000000002083

**Published:** 2024-09-11

**Authors:** Yeongyun Jung, Ryeong-Hui Kim, Eun Kyung Lee, Cheong Hoon Seo, So Young Joo, Jae-Ho Shin, Yoon Soo Cho

**Affiliations:** a Burn Institute, Hangang Sacred Heart Hospital, Hallym University College of Medicine; b Department of Rehabilitation Medicine, Hangang Sacred Heart Hospital, Hallym University College of Medicine, Seoul; c NGS Core Facility, Kyungpook National University, Daegu, Republic of Korea

**Keywords:** burn scars, extracorporeal shock wave therapy, microbial community structure, microbial diversity, skin microbiome

## Abstract

**Background:**

The effectiveness of extracorporeal shock wave therapy (ESWT) has been demonstrated in various medical fields, including burn medicine. It promotes wound healing, improves blood flow, and modulates the inflammatory responses. The recovery speed and outcomes of skin diseases are influenced by the skin microbiome; however, studies examining the effects of specific treatments on the skin microbiome are lacking. This study investigated the impact of ESWT on the skin microbiome of burn patients, focusing on the microbial diversity and community structure within burn scars.

**Materials and methods:**

In the retrospective case–control study, 19 patients with burn scars were treated with ESWT, and changes in their skin microbiome were evaluated. ESWT was administered weekly for 3 months, and samples were collected from the ESWT-treated burn scars and untreated normal skin. Blood chemistry, and pain and itching scores were evaluated during sample collection. The collected samples were then subjected to 16S rRNA sequencing. Microbial community analysis was conducted using the QIIME2 and R packages.

**Results:**

After ESWT, changes in alpha diversity indices were observed in burn scars. Faith phylogenetic diversity (*P*<0.05) and observed features (*P*<0.01) increased, whereas the evenness index decreased (*P*<0.01); no marked changes were noted in untreated skin. Beta diversity analysis showed stable microbial community structures in both the treated and untreated areas. A considerable increase in *Micrococcus* and *Staphylococcus* abundance was observed. Network analysis revealed a more open microbial network structure after ESWT, indicating adaptive changes in the microbial community.

**Conclusion:**

ESWT enhances microbial diversity and modifies microbial community structure in burn scars, promoting a more balanced and functionally supportive microbiome. ESWT aids in scar remodeling and positively influences skin microbiome dynamics, contributing to improved skin health and recovery.

## Introduction

Highlights

- Skin health depends on the composition and diversity of the microbiome in the skin.
- Extracorporeal shock wave therapy (ESWT) leads to increased alpha diversity in burn scars.
- ESWT does not disrupt the overall microbial balance of the skin microbiome in burn patients.
- ESWT modifies the scar microbiome network, resulting in a more open structure.

The cutaneous microbiome refers to a diverse community of microorganisms that resides on the skin. A balanced microbiome plays a crucial role in the maintenance of skin health by reinforcing the skin barrier, protecting against external environmental stimuli, and modulating immune responses through intercellular signaling^1–3^. Moreover, certain skin microorganisms produce antibacterial substances or inhibit the growth of pathogens, thereby preventing skin infections and managing the inflammatory responses^1,2,4^. Imbalances in the skin microbiome can disrupt protective mechanisms, potentially leading to various dermatological conditions, including inflammation, infections, atopic dermatitis, and acne. Distinct differences in microbiome composition between individuals with skin diseases, such as atopy and psoriasis, and those with healthy skin have been noted, with specific microbiomes implicated in the pathogenesis of these conditions^4,5^. Furthermore, it has been reported that patients with burns in the subacute phase who have completed re-epithelialization of burn wound show dysbiosis in both affected and nonaffected skin^6^. Thus, preserving the equilibrium of the skin microbiome is crucial in preventing these skin disorders^5,7^.

10–91% of burn injuries is followed by hypertrophic formation of postburn scars^8,9^, which can result in pain, itching, and functional problems in the patients with burns^10^. Despite various treatment methods, including skin rehabilitation therapy, compression garments, silicone gel or sheets, and intralesional injections^11^, preventing scar complications remains an issue that needs to be resolved. Recently, extracorporeal shock wave therapy (ESWT) has conducted to decrease pain, itching, and hypertrophic scarring in patients with burns^12–16^.

ESWT is a noninvasive treatment that utilizes high-amplitude sound waves that reverberate three-dimensionally to treat various musculoskeletal conditions. According to previous studies, this treatment method improves the wound healing process by promoting cell proliferation and migration, improving blood flow to the wound area by increasing the creation of new blood vessels, and suppressing inflammatory responses by reducing the production of inflammatory mediators^17,18^. Initially, ESWT was prominent in orthopedics and urology; its application has broadened to include burn medicine, and more recently, esthetic medicine and dermatology. This adoption across specialties underscores the versatility and efficacy of this treatment modality^17,18^. ESWT has been shown to alleviate pain and itchiness associated with burn scars by regulating neurotransmitter activity^12,13^. Moreover, it reduces the hypertrophic response in burn scars, inhibits the activation of fibroblasts and keratinocytes, and modulates collagen production^14,15,19^. In addition, the application of ESWT to the remodeling stage of scars that had undergone split-thickness skin grafting (STSG) using an artificial dermis promoted the production of sebaceous glands and improved scar appearance^16^.

Recent research has focused on observing changes in the skin microbial community following treatment of certain skin diseases, including atopic dermatitis and seborrheic dermatitis. These studies aimed to analyze the influence of these changes on disease progression and outcomes^20^. Although studies like these have elucidated the effects of treatments on the skin microbiome, there is little research examining the influence of any treatment on the skin microbiome in patients with burns. Therefore, this study aimed to show the effect of ESWT on the skin microbiome of patients who had undergone ESWT for burn scars using next-generation sequencing. The results of this study can suggest the applicability of ESWT as a therapeutic alternative to induce microbial balance, not only in burn scars, but also in various skin diseases that have been found to be related to the skin microbiome.

## Materials and methods

### Patient enrollment and data collection

This is a retrospective case–control study to use the samples collected from burn patients in the previous study that researched the microbiome of burn scars^21^, which was conducted between January 2020 and July 2023, approved by the Institutional Review Board of Hangang Sacred Heart Hospital (HG2020-007), and written informed consent was obtained from all participants. Pre-existing samples, which had been collected to analyze the scar microbiome from 40 patients with burns between January 2022 and June 2023, were utilized for this analysis. Among these 40 patients, 19 patients who underwent ESWT were enrolled in the retrospective study. All participants were inpatients receiving burn rehabilitation therapy at the Department of Rehabilitation Medicine. Because the inclusion and exclusion criteria of the previous study had been applied, they had no skin diseases such as dermatitis and psoriasis, no metabolic diseases such as diabetes, and were not receiving oral or topical antibiotic treatment. All of them had been previously advised about scar management and applied the O Medical Device moisturizers to burn scars and control normal skin two to three times daily.

Clinical characteristics such as age, sex, and total body surface area (%TBSA) were extracted from the electronic medical records and entered into a database. Blood samples for blood chemistry analysis were collected after fasting for at least 8 h.

### Ethics statement

The study design and protocol were approved by the Institutional Review Board of Hangang Sacred Heart Hospital (HG2024-005) and registered with the Clinical Research Information Service (KCT0009421). The requirement for the acquisition of written informed consent from the study subjects was waived owing to the nature of our study, which was that this used the samples had collected with proper consent and IRB approval (HG2020-007) for microbiome research. All procedures were conducted in accordance with the relevant guidelines and regulations.

### ESWT procedure

ESWT was performed using the Duolith SD-1 device (StorzMedical), which utilizes an electromagnetic cylindrical coil source to focus shock waves. Treatments on the scarred tissue were administered at an intensity of 100 impulses/cm², energy flux density of 0.05–0.20 mJ/mm², and frequency of 4 Hz. Each session involved delivery of 1000–2000 pulses, with a total of 12 sessions conducted at weekly intervals for 3 months.

### Sample collection

In our previous study^21^, untreated (normal skin that had not been burned) and treated (burn scar that underwent ESWT) skin samples were collected from the same patients to control for the variables associated with burns. Sampling was performed using sterile swabs moistened with saline on the day of admission (0M; pretreatment) and again 3 months later (3M; post-treatment). The collected samples were stored at −80°C until DNA extraction.

Each person harbors a unique microbial community, and the biomechanical properties of burn scars are influenced by various factors such as burn depth, burn type, and genetic differences. This high degree of variability makes it challenging to distinguish changes in the microbiome attributable to ESWT from those resulting from these intrinsic factors. Therefore, the same patient’s normal skin was used as a control before and after ESWT treatment to minimize the variability introduced by differences between individuals.

### DNA extraction and sequencing

DNA was extracted using the DNeasy PowerSoil Pro Kit (Qiagen) according to the protocols established in a previous study^21,22^. The 16S rRNA gene, specifically from the V4 to V5 hypervariable regions, was amplified under the conditions described previously^21,22^. Sequencing was performed using the Illumina MiSeq platform at the KNU NGS Core Facility, Kyungpook National University, South Korea, using the MiSeq Reagent Kit v3 for 300 bp paired-end reads.

### Microbiome analysis

DNA was extracted from the collected samples and sequenced using the Illumina MiSeq platform at the KNU NGS Core Facility, Kyungpook National University, South Korea, focusing on 16S rRNA gene sequencing. Skin microbiota analysis was conducted using QIIME2 (v2023.9) software with the Deblur denoising tool^23,24^. Initial steps involved the removal of primers and adapters using the q2-cutadapt plugin, followed by quality filtering of sequences with the q2-quality-filter plugin. The q2-deblur plugin was used for the sequence denoising. Taxonomic classification of the sequences was performed using the q2-feature-classifier plugin with sklearn against the SILVA (v138) and Greengenes2 (v2022.3) databases^25,26^. Phylogenetic trees (both rooted and unrooted) were constructed using the mafft, mask, and FastTree protocols within QIIME2, and these trees were used in subsequent diversity analyses.

### Statistical analysis of clinical and microbiota diversity

Clinical parameters were assessed using paired *t*-tests at designated intervals. Changes in the alpha diversity of the microbiota within normal skin and scars were evaluated using the Wilcoxon matched-pairs signed-rank test. Beta diversity was analyzed based on both weighted and unweighted UniFrac distances using the QIIME2 (v2023.9) software for calculation. The differences between pre-extracorporeal and postextracorporeal shock wave therapy (ESWT) samples were analyzed using permutational multivariate (PERM) analysis of variance (ANOVA).

### Microbial abundance and network analysis

Relative abundance assessments and network analyses were conducted in R (v4.2.1), using comprehensive data analysis packages such as ‘microbiome’^27^, ‘dplyr’^28^, ‘phyloseq’^29^, ‘hrbrthemes’^30^, ‘gcookbook’^31^, ‘tidyverse’^32^, and ‘qiime2R’^33^. Bacterial genera associated with ESWT-treated scars were identified using the linear discriminant effect size (LEfSe) analysis^34^. Network associations were examined using a permutation test with 1000 iterations using the NetCoMi package in R^35^. The resultant data were visually represented through figures and graphs created in R using the ‘ggplot2’ and ‘ggpubr’ packages^36^.

### Functional pathway analysis

Functional predictions were made using the PICRUSt2 tool^37^, facilitated by creating copy number count tables with the q2-picrust2 plugin in QIIME2. Analysis of Kyoto Encyclopedia of Genes and Genomes (KEGG) orthologs (KO) included normalization of expression levels using median values between the treated and untreated samples. Changes in expression were quantified using log_2_ (fold change), with statistical significance assessed using *t*-tests and Wilcoxon matched-pairs signed-rank tests. Visualization of the results, including volcano and bar plots, was performed using the ‘ggplot2’ package in R^36^.

## Results

### Characteristics between treated and untreated samples

Nineteen patients were enrolled in this study. The baseline demographics are presented in Table 1 and Supplementary Table 1 (Supplemental Digital Content 1, http://links.lww.com/JS9/D422). The average age was 45 years, with males constituting 52.63% of the cohort. Third-degree burns were the most common, affecting 68.42% of patients. A total of 76 samples (treated: *n*=38; untreated: *n*=38) were obtained pretreatment and post-treatment. The mean collection time was 99.53 days. The mean burned body surface area (BSA) was 14.16%. Scar severity was assessed using the Vancouver Scar Scale, with an average score of 8.26, and the observer and patient scar assessment scales, with average scores of 23.84 and 26.58, respectively. Upon comparing the clinical characteristics of burn scars before and after ESWT, the erythrocyte sedimentation rate was found to decrease (*P*<0.05), along with the Numerical Rating Scales for pain (*P*<0.001) and itch (*P*<0.001; Table 2).

**Table 1:** Demographic characteristics of the patients enrolled in this study.

Variables	Patients (*n*=19)
Age (years)	45.37±9.48
Sex	
Male	10 (52.63%)
Female	9 (47.37%)
Burn degree	
Superficial 2nd degree	3 (15.79%)
Deep 2nd degree	3 (15.79%)
3rd degree	13 (68.42%)
Time after injury (days)	99.53±95.66
Burned BSA, %	14.16±13.32
VSS total score	8.26±1.97
OSAS total score	23.84±7.63
PSAS total score	26.58±10.65

Data are expressed as mean±SD for continuous variables and as numbers (percentages) for categorical variables.

BSA, body surface area; OSAS, Observer Scar Assessment Scale; PSAS, Patient Scar Assessment Scale; VSS, Vancouver Scar Scale.

**Table 2:** Comparison of clinical parameters pre-extracorporeal and postextracorporeal shock wave therapy.

	Time of sample collection		
Variables	0M (*n*=38)	3M (*n*=38)	*P*
ESR, mm/H	14.21±10.70	8.58±5.92	0.015
CRP, mg/l	1.98±3.49	2.16±5.65	0.906
Numerical rating scale			
Pain	5.84±2.59	2.79±1.93	< 0.001
Itch	4.32±2.36	2.05±1.62	< 0.001

Data are expressed as mean±SD. Paired *t*-tests were used to analyze the data.

0M, pretreatment; 3M, three months post-treatment; CRP, C-reactive protein; ESR, erythrocyte sedimentation rate.

### Comparison of microbiome diversity in treated and untreated samples

**Figure 1:**
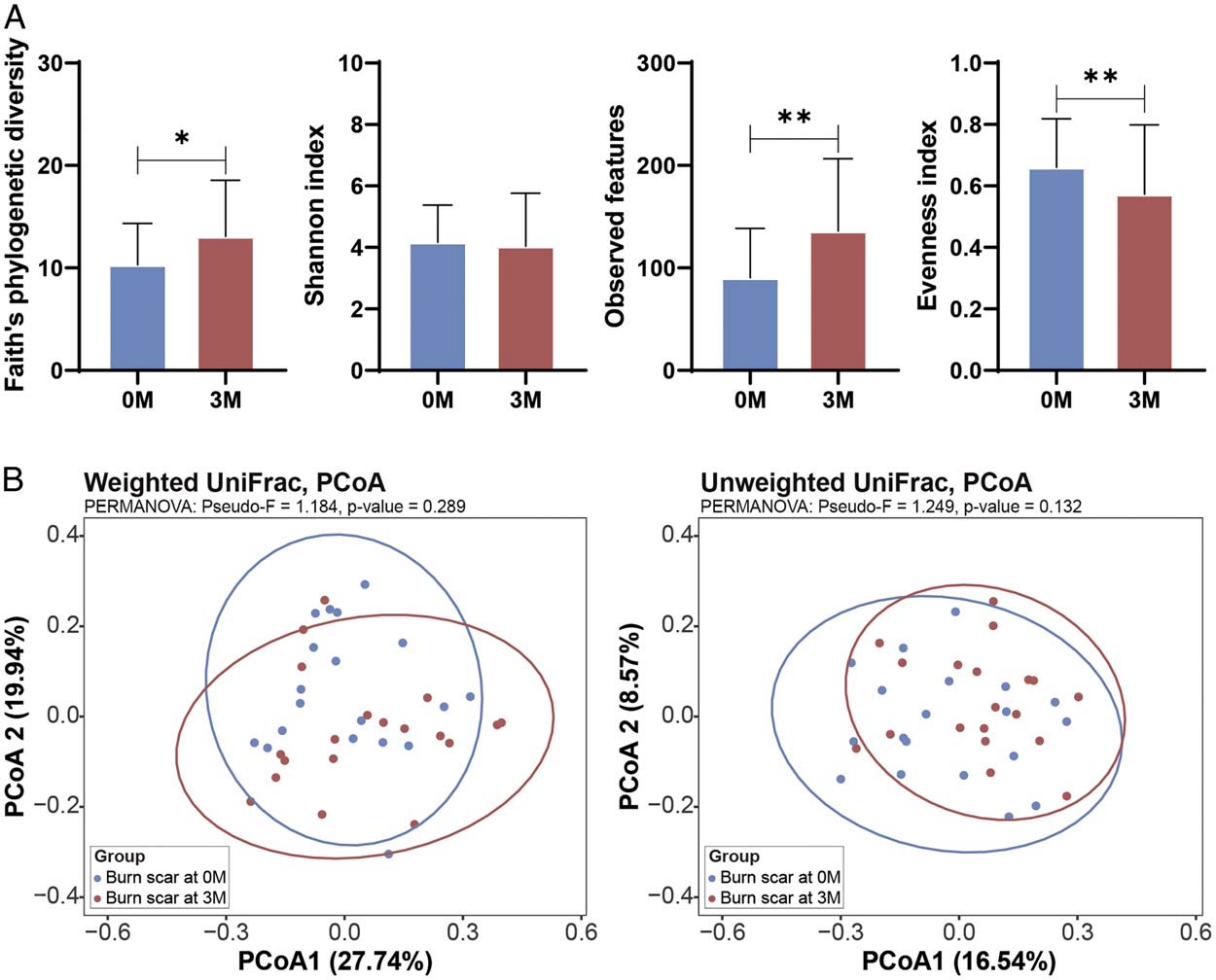
Changes in bacterial diversity in burn scars pre-ESWT and post-ESWT. (A) Alpha diversity measures of the burn scar microbiome at baseline (0 M) and after 3 months (3 M). The indices included Faith’s phylogenetic diversity, the Shannon index, observed features, and evenness index. * *P*<0.05 and ** *P*<0.01 are considered statistically significant. (B) The beta diversity of the microbiome was analyzed using weighted and unweighted UniFrac distances depicted in PCoA plots. The ellipses represent 95% CI around the centroid of each group. ESWT, extracorporeal shock wave therapy; PCoA, principal coordinate analysis.

**Figure 2:**
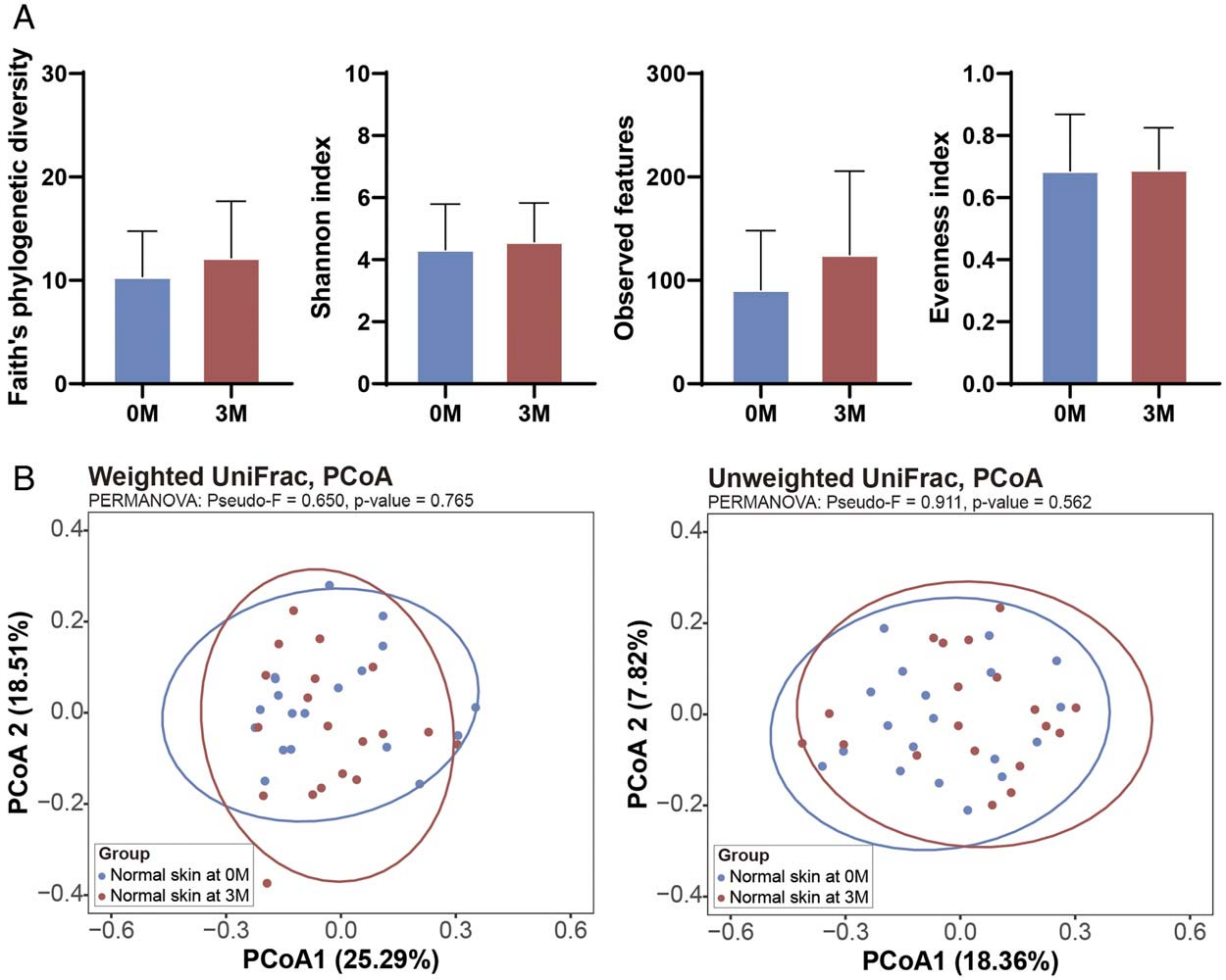
Analysis of microbial diversity in untreated skin pre-ESWT and post-ESWT. (A) Alpha diversity measures of the burn scar microbiome at baseline (0M) and after 3 months (3M). The indices included Faith’s phylogenetic diversity, the Shannon index, observed features, and evenness index. * *P*<0.05 and ** *P*<0.01 are considered statistically significant. (B) The beta diversity of the microbiome was analyzed using weighted and unweighted UniFrac distances depicted in PCoA plots. The ellipses represent 95% CI around the centroid of each group. ESWT, extracorporeal shock wave therapy; PCoA, principal coordinate analysis.

Sequencing depth analysis indicated that all samples reached a plateau on the rarefaction curves (Supplementary Figure 1, Supplemental Digital Content 2, http://links.lww.com/JS9/D423), confirming that the sequencing data were adequate for comprehensive analysis. In the treated samples, changes were observed in alpha diversity metrics within the burn scars post-treatment (*P*<0.05). In contrast, the Shannon diversity indices were similar, other alpha diversity measures, such as Faith’s phylogenetic diversity and observed features showed increases from pretreatment to post-treatment (*P*<0.05; Fig. 1A). Additionally, the evenness index decreased post-treatment (*P*<0.05), indicating an increase in the dominance of certain microbes within the treated scar. In contrast, the untreated samples exhibited no changes in either alpha or beta diversity over the 3M period (*P*>0.05; Fig. 2). This suggests that although ESWT can induce substantial shifts in the microbial composition of burn scars, it does not affect the microbial diversity of untreated skin areas.

Beta diversity analysis of both treated and untreated samples revealed no marked changes pretreatment and post-treatment (Fig. 1B). This suggests that the overall microbial community structure maintains stability in terms of species interactions and community assembly. These findings indicate that ESWT specifically influences microbial dynamics within treated areas, potentially contributing to altered microbial functions important for scar modification and healing processes, while not affecting the microbial ecosystem of untreated skin.

### Changes in microbiome diversity in treated and untreated samples

**Figure 3:**
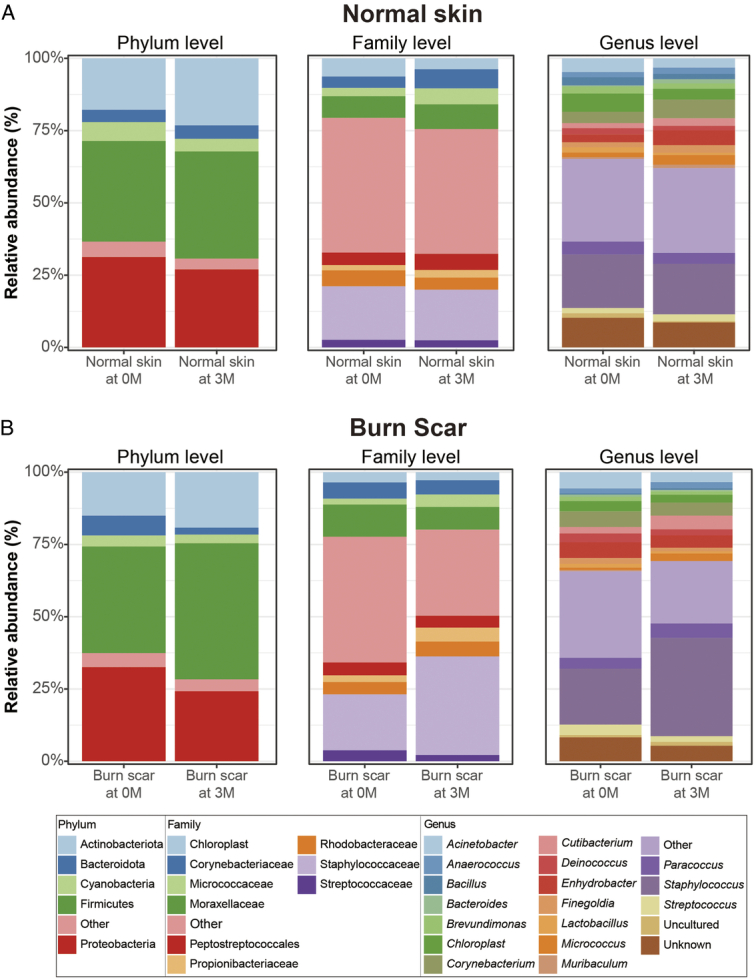
Relative abundance of bacteria at the phylum, family, and genus level. (A) Untreated samples and (B) treated samples at 0 months (0M) and 3 months (3M), without and with extracorporeal shock wave therapy. ‘Others’ denotes bacterial taxa with an abundance of ≤0.5% at the phylum and family levels, or ≤0.05% at the genus level, and a prevalence of ≤50%. ‘Unclassified’ includes sequences that could not be classified to any known taxonomic category.

This study tracked alterations in the microbiome composition at the phylum, family, and genus levels over three months in both ESWT-treated burn scars and untreated normal skin (Fig. 3, Supplementary Figure 2, Supplemental Digital Content 2, http://links.lww.com/JS9/D423, and Supplementary Table 2, Supplemental Digital Content 3, http://links.lww.com/JS9/D424). The untreated samples showed no marked shifts in dominance order among the top three constituents at any taxonomic level over the 3-month period (Fig. 3A). In contrast, the treated samples exhibited notable shifts in diversity. At the phylum level, the proportions remained consistent throughout the treatment period, with firmicutes (0M: 36.87%; 3M: 47.10%), proteobacteria (0M: 32.53%; 3M: 24.20%), and actinobacteria (0M: 15.02%; 3M: 19.19%) dominating. At the family level, the most abundant group at 0M was composed of compromised less dominant taxa (other) with abundances below 0.5% and prevalence below 50% (43.50%), and an increase in the dominance of *Staphylococcaceae* (34.13%; *P*<0.05) occurred at 3M. A similar trend was observed at the genus level, where data from 0M showed a prevalence of fewer dominant taxa (other; 28.00%); however, post-treatment results revealed an increase in *Staphylococcus* (33.99%; *P*<0.05).

**Figure 4:**
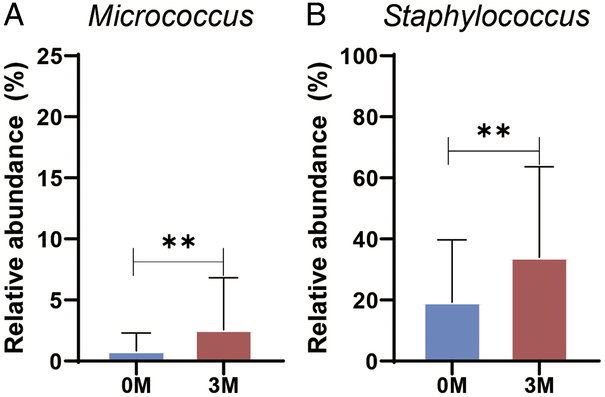
Impact of ESWT on the relative abundance of bacterial populations in burn scars. (A) Relative abundance of *Micrococcus* and (B) *Staphylococcus* measured before (0M) and three months after (3M) ESWT. **P<0.01 is considered statistically significant. ESWT, extracorporeal shock wave therapy.

**Figure 5:**
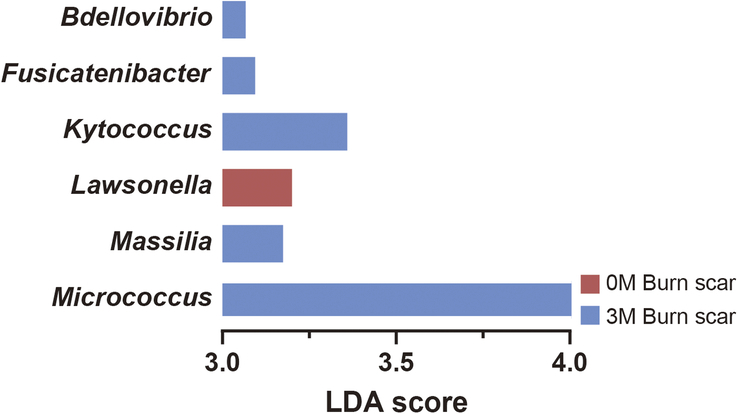
Differential abundance of microbial genera in burn scars analyzed using LDA LEfSe measurements. The LEfSe measurements were used to identify differentially abundant microbial genera in burn scars before (0M) and after extracorporeal shock wave therapy (3M). The LDA score indicates the effect size of each genus. Genera shown in red were more abundant at baseline (0M), whereas those in blue were more abundant at 3 months (3M). Only genera meeting a significant LDA threshold score (> 3.0) are displayed. LDA, linear discriminant analysis; LEfSe, LDA combined with effect size.

Comparative analysis of genus-level changes at 0M and 3M highlighted increases in *Micrococcus* and *Staphylococcus* (*P*<0.05; Fig. 4). LEfSe was used to comprehensively analyze these shifts (Fig. 5, Supplementary Table 3, Supplemental Digital Content 3, http://links.lww.com/JS9/D424). *Lawsonella* was the dominant genus in the samples at 0M, whereas *Micrococcus, Kytococcus, Massilia, Fusicatenibacter*, and *Bdellovibrio* were predominant at 3M. These findings suggest that ESWT affects the settlement and proliferation of microbial community members, particularly aiding in the dominance of *Staphylococcus* and *Micrococcus* in treated burn scars.

### Impact of ESWT on microbial network dynamics in burn scars

**Figure 6:**
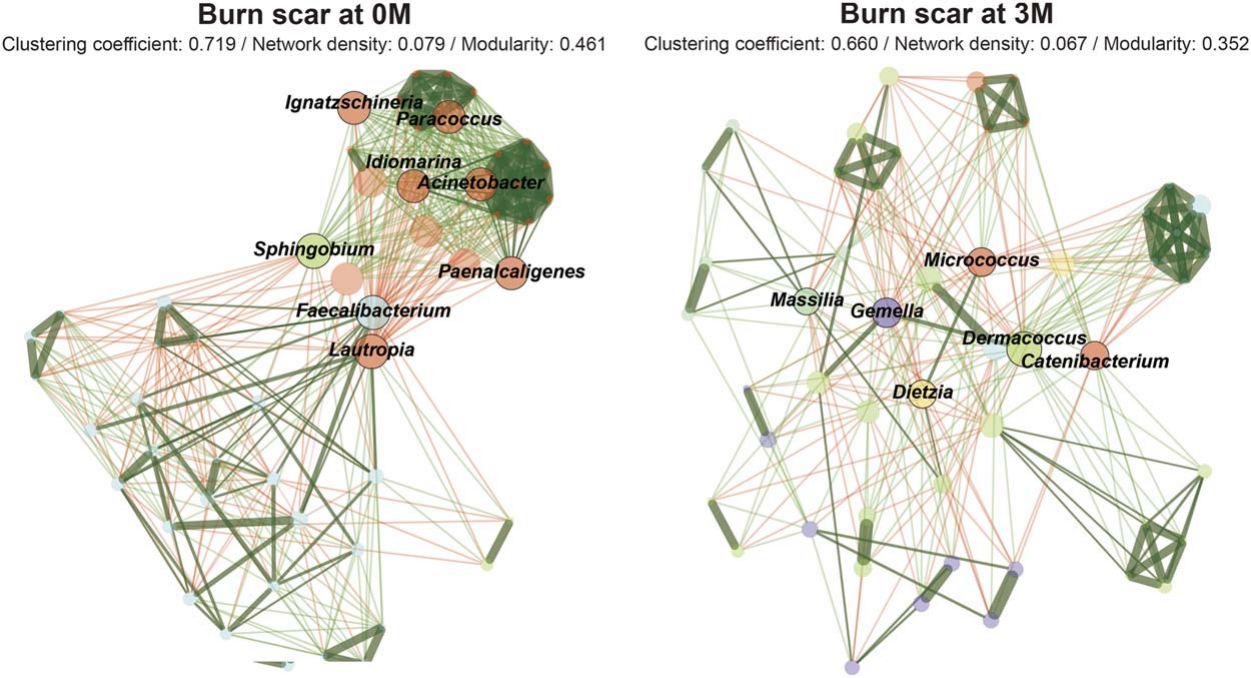
Network analysis of the skin microbiome at the bacterial ASV level. The ASV is depicted as a node whose size is proportional to the over-abundance relative to background. Nodes are sized and colored according to normalized counts and cluster membership. Colors are automatically selected by the software, NetCoMI. Edge color reflects the correlation among nodes (green and red for positive and negative correlations, respectively). ASV, amplicon sequence variant.

Through network analysis, the relationships among amplicon sequence variants (ASVs) pre-ESWT and post-ESWT (Fig. 6) were investigated. Marked changes were observed in the microbial network metrics of the treated samples. The clustering coefficient decreased from 0.719 to 0.660, indicating that the microbial community is less tightly interconnected. The network density was reduced from 0.079 to 0.067, suggesting fewer interactions among the microbial species. Furthermore, modularity, which measures the degree to which a network can be divided into clearly defined modules, decreased from 0.461 to 0.352. This indicated a shift towards a less compartmentalized microbial network following ESWT.

Pretreatment, the hub microbes in the scars included ignatzschineria, paracoccus, idiomarina, acinetobacter, sphingobium, paenalcaligenes, faecalibacterium, and lautropia. However, post-treatment, new hubs emerged, such as micrococcus, massilia, gemella, dermacoccus, catenibacterium, and dietzia. this transition suggests that ESWT influences the microbial structure and connectivity, reflecting an adaptive phase where the microbial community stabilizes into a new equilibrium that is more suited to the altered skin environment post-treatment. These findings highlight the potential of ESWT to affect the structural dynamics of the skin microbiome, thereby fostering a community composition that may improve skin healing and resilience.

### Predicted functional changes in the burn scar microbiome following ESWT

**Figure 7:**
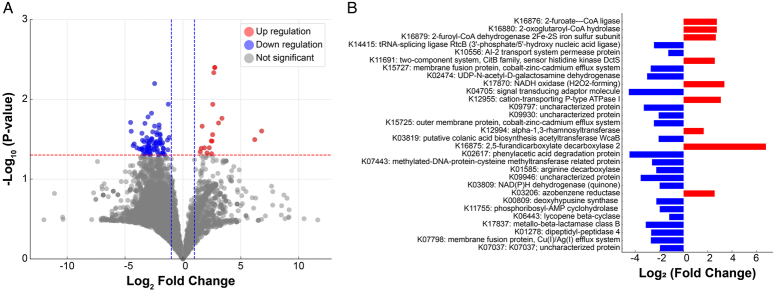
Comparison of functional profiles in burn scars pre- and post-extracorporeal shock wave therapy using PICRUSt predictions. (A) Volcano plot based on normalized expression data. Significantly upregulated KOs are denoted by red circles (*n*=19), downregulated KOs with blue circles (*n*=81), and KOs not showing statistical differences, with gray circles (*n*=7371). The vertical axis represents the -log_10_ (*P*-value), and the horizontal axis represents the log2 (fold change). The KOs with significant expression changes were distinguished based on a *P*-value <0.05 and an absolute fold change greater than 2. (B) Bar chart of the top 30 KOs with significant expression changes. KOs with increased expression are colored in red, and those with decreased expression are colored in blue. Each bar is annotated with a functional description. Kyoto Encyclopedia of Genes and Genomes (KEGG) orthologs (KO).

All the predicted KOs are listed in Supplementary Table 4 (Supplemental Digital Content 3, http://links.lww.com/JS9/D424). A total of 19 and 81 KOs were upregulated and downregulated, respectively (*P*<0.05). An additional 7371 KOs remained unchanged (*P*>0.05; Fig. 7A). The top 30 KOs with changes in expression were identified (*P*>0.05; Fig. 7B). Genes involved in metabolic pathways and environmental response mechanisms, such as 2-furoate-CoA ligase (*hmfD*; EC: 6.2.1.31), and sensor histidine kinase (*dctS;* EC: 2.7.13.3), exhibited increased expression levels post-treatment. This upregulation suggests that the microbial ability to adapt and signal in response to environmental changes induced by ESWT is enhanced.

Conversely, genes critical for stress responses and nutrient transport, such as tRNA-splicing ligase (*RtcB*; EC: 6.5.1.8), and the cobalt–zinc–cadmium efflux system (*czcB*), were downregulated (*P*<0.05). This reduction in expression may imply a potential suppression of specific microbial defense mechanisms, which could alter the resilience of the skin microbiome and its interactions with the host environment. Despite these changes, the vast majority of the functional capacities of the skin microbiomes, as reflected by the stable expression of a substantial number of KO genes, suggest that the core functional integrity of the microbiome was maintained post-ESWT. This indicates that, while ESWT leads to changes in certain microbial functions, the overall microbial framework remains stable, ensuring continued support for skin health and recovery.

## Discussion

The microbiome exerts a profound impact on host health, which is vital, not only for managing chronic diseases, but also for maintaining overall skin health^38–41^. The 16s rRNA amplicon sequencing was performed on skin microbial samples collected from patients with burns pretreatment and post-treatment. The study detected marked changes in the microbial community of the scar area post-treatment, most notably an increase in alpha diversity and changes in microbial community composition. This finding indicates that ESWT can increase the expression of growth factors that promote wound healing and vascular formation. EWST suppresses inflammatory response by reducing the production of inflammatory mediators^17,18^. It has also been reported that ESWT can affect skin biomechanical properties^16,19^. This study is the first of its kind to explore changes at the microbiological level using ESWT and elucidates the effects of ESWT.

In this study, we showed the effects of ESWT using samples obtained from the same patients at 0 months and 3 months from ESWT-treated or untreated areas. The human microbiome is highly individualized, with each person harboring a unique microbial community. This individuality can significantly impact the outcomes of microbiome studies^42,43^. Additionally, the biomechanical properties of burn scars are influenced by various factors such as burn depth, burn type, and genetic differences. It is known that burn patients’ characteristics, such as total body surface area and ICU admission, as well as biomechanical properties like trans-epidermal water loss, can affect the microbial community^21^. For these reasons, the normal skin of the same patient (specifically from the contralateral site of the burn scar) was used as a control instead of randomizing different individuals as the control group. In this case, the question arises as to how changes in the scar microbiome after ESWT can be distinguished from natural regenerative changes in the scar. Subacute burn patients, whose re-epithelialization of burn wounds has been completed, have been reported to exhibit microbial imbalances in both affected and unaffected skin areas compared to patients without burns and no difference of skin microbiome between affected and unaffected skin areas^6^. Thus, we used the same patient’s normal skin as a control to compare the skin microbiome before and after ESWT treatment. This methodology minimized interindividual variability and established a clear baseline.

It is known that a higher biodiversity reflects a healthier microbial ecosystem^44–46^. The skin microbiome is shaped by the skin microenvironment, with bacteria obtaining nutrients from the stratum corneum, sebaceous glands, and eccrine and apocrine secretions^47^. The current study indicates that the alpha diversity of the microbial community in burn scars increased after ESWT. Therefore, the increase in alpha diversity was presumed to result from biophysical changes in the skin induced by ESWT, such as collagen and sebaceous gland production^16,19^. Sebum, in particular, plays a crucial role in skin health by providing moisturization, forming a hydrophobic protective layer, and serving as a source of lipids for the microbial flora^48^. The free fatty acids produced by the hydrolysis of these lipids promote the adhesion of symbiotic bacteria and lower the pH of the skin, thereby inhibiting the growth of pathogenic microorganisms^48^. Therefore, increasing alpha diversity through ESWT in burn scars is expected to have a positive effect on the maintenance of skin microbiome balance. Beta diversity was used to measure the changes in microbial species composition between different samples^49^. Beta diversity did not show significant changes in either ESWT-treated or untreated areas. This indicates that although ESWT enriches the local microbial community, it does not disrupt the overall microbial balance.

This study showed that the application of ESWT induced substantial changes in skin microbiota composition, as there was an increase in *Staphylococcus* populations. These microorganisms are typical commensals of the skin^50^. Within the *Staphylococcus* genus, species such as *S. aureus* are known to develop antibiotic resistance and adversely affect skin health^51^. Conversely, species such as *S. epidermidis*, *S. hominis*, and *S. lugdunensis* produce various peptides that inhibit the growth of *S. aureus* ^1^ *. Staphylococcus* is known to strengthen skin barrier function and regulate immune responses, which are important for maintaining skin health. Studies have shown that *S. epidermidis* produces antimicrobial peptides that compete with harmful bacteria, protect the skin, promote a healthy microbial balance, and prevent skin infections^1,52,53^. Previous studies have reported that ESWT increases collagen levels^19^ and reduces fibronectin in fibroblasts derived from scar tissue^14^. *S. epidermidis* colonizes the skin by interacting with type I collagen^54^. However, *S. aureus* is known to have a higher binding affinity for fibronectin than for collagen^55^. For this reason, the increased *Staphylococcus* is predicted to be *S. epidermidis*. Moreover, the findings demonstrate that ESWT can affect microbial community composition; an altered microbial community is expected to have positive effects on skin health, including improved barrier protection and immune function.

Network analyses serve as effective methods for identifying complex biological systems characterized by intricate interactions among members, such as microbial communities^56^. According to the network analysis used in this study, the clustering coefficient decreased. This decrease indicates a reduced robustness of interconnections within the microbial community, suggesting a more open network structure that can promote new interactions and pathways for microbial activity. Additionally, reduced network density and modularity imply a less compartmentalized microbial environment. This more integrated network arrangement potentially allows for a more cooperative microbial environment, which is important for the dynamic adaptation to environmental changes following treatment. Moreover, changes in the microbiome represent a substantial reorganization of key microbial players, which may influence the metabolic profile and functional capacity of the skin microbiome.

Although this study provides information on the effects of ESWT on the skin microbiome of burn scars for the first time, it has several limitations. One major limitation of this study is the scope of microbial analysis, which was limited to 16S rRNA gene sequence analysis^57^. This sequencing method, while effective in identifying microbial communities up to the genus level, has limitations in distinguishing microorganisms at the species or strain level. Additionally, the short follow-up period post-treatment in our study restricted our ability to assess the sustainability of microbiome changes over time. A longer follow-up period would provide a more accurate measurement of the long-term effects of ESWT on skin recovery and health. Furthermore, the study was conducted using a small sample size, which may limit the generalizability of our findings. Another limitation is the use of nonspecific inflammatory markers. Erythrocyte sedimentation rate and C-reactive protein are commonly used general inflammation markers, but they lack sensitivity or specificity^58^. The pathophysiology of burn injuries is very complex and involves a variety of inflammatory biomarkers^59^. Therefore, if more diverse inflammatory biomarker data had been used, the impact of ESWT treatment on the patient’s inflammatory state could have been evaluated more accurately.

## Conclusions

In conclusion, the findings of this study demonstrate that ESWT increases alpha diversity in burn scars and can affect microbial communities and network composition by increasing skin commensals, which may benefit skin health. Furthermore, ESWT not only has a positive effect on scar remodeling during burn scar recovery but may also have an indirect effect on skin health and recovery processes by influencing the skin microbiome. To elucidate the effect of ESWT on the composition and functional capacity of microbial communities, future studies should employ long-term shotgun metagenomic sequencing with substantially larger sample sizes and be conducted with randomized controlled trials including patients with ESWT and patients without ESWT. These follow-up studies will elucidate how ESWT influences the skin microbiome over an extended period of time.

## Ethical approval

The study protocol was approved by the Institutional Review Board and Ethics Committee of Hangang Sacred Heart Hospital. (approval no. HG2024-005).

## Consent

The requirement for the acquisition of written informed consent from the study subjects was waived owing to the nature of our study, which was that this used the samples had collected with proper consent and IRB approval (HG2020-007) for microbiome research. All procedures were conducted in accordance with the relevant guidelines and regulations.

## Source of funding

This study was supported by the Basic Science Research Program through the National Research Foundation of Korea (NRF) funded by the Ministry of Education (2020R1l1A3074150 and RS-2023- 00272448).

## Author contribution

Y.J. and Y.S.C.: conceived and designed the study; E.K.L.: collected and assembled the data; R.H.K.: performed the experiments; Y.J.: analyzed and interpreted the data; Y.J. and Y.S.C.: wrote the manuscript; C.H.S., S.Y.J., J.H.S., and Y.S.C.: revised the manuscript. All authors have reviewed the results and approved the final version of the manuscript.

## Conflicts of interest disclosure

The authors declare no conflicts of interest.

## Research registration unique identifying number (UIN)

1. Name of the registry: The Clinical Research Information Service (cris.nih.go.kr), Korean Center for Disease Control and Prevention, Ministry of Health and Welfare, Osong, Republic of Korea).

2. Unique identifying number or registration ID: KCT0009421.

3. Hyperlink to your specific registration (must be publicly accessible and will be checked): https://cris.nih.go.kr/cris/search/detailSearch.do?seq=27078&status=1&seq_group=27078&search_page=M.

## Guarantor

Yoon Soo Cho.

## Data availability statement

The datasets generated and/or analyzed in the current study are available in the NCBI Sequence Read Archive database (accession number: PRJNA1108912).

## Provenance and peer review

This paper was invited by Dr Kandiah Raveendra, Guest Editor for the special issue on Shock wave treatment.

## Supplementary Material

SUPPLEMENTARY MATERIAL
