# Supplementary material for: Effect of extracorporeal shock wave therapy on the microbial community in burn scars: retrospective case–control study
Source: Int J Surg. 2024 Sep 11;110(12):7477–86. doi: 10.1097/JS9.0000000000002083 (PMC11634101; doi:10.1097/JS9.0000000000002083)
Supplement: SUPPLEMENTARY MATERIAL [file js9-110-7477-s001.docx]

**Supplementary table 1. Demographic characteristics of the patients enrolled and excluded from this study**

| **Variables** | | **Enrolled Patients (n = 19)** | **Excluded Patients (n = 21)** |
| --- | --- | --- | --- |
| **Age (years)** | | 45.37 ± 9.48 | 48.24 ± 10.76 |
| **Sex** | Male | 10 (52.63%) | 10 (48.24%) |
|  | Female | 9 (47.37%) | 11 (52.38%) |
| **Burn degree** | Superficial 2^nd^ degree | 3 (15.79%) | 2 (9.52%) |
|  | Deep 2^nd^ degree | 3 (15.79%) | 7 (33.33%) |
|  | 3^rd^ degree | 13 (68.42%) | 12 (57.14%) |
| **Time after injury (days)** | | 99.53 ± 95.66 | 128.67 ± 72.15 |
| **Burned BSA, %** | | 14.16 ± 13.32 | 22.33 ± 18.52 |
| **VSS total score** | | 8.26 ± 1.97 | 8.38 ± 2.28 |
| **OSAS total score** | | 23.84 ± 7.63 | 24.00 ± 8.34 |
| **PSAS total score** | | 26.58 ± 10.65 | 29.81 ± 10.24 |

Data are expressed as mean ± standard deviation for continuous variables and as numbers (percentages) for categorical variables.

Abbreviations: BSA, body surface area; VSS, Vancouver Scar Scale; OSAS, Observer Scar Assessment Scale; PSAS, Patient Scar Assessment Scale
