# Supplementary material for: Effect of extracorporeal shock wave therapy on the microbial community in burn scars: retrospective case–control study
Source: Int J Surg. 2024 Sep 11;110(12):7477–86. doi: 10.1097/JS9.0000000000002083 (PMC11634101; doi:10.1097/JS9.0000000000002083)
Supplement: SUPPLEMENTARY MATERIAL [file js9-110-7477-s002.docx]

**Supplemental Digital Content**

**Title: Effect of Extracorporeal Shock Wave Therapy on the Microbial Community in Burn Scars**

**
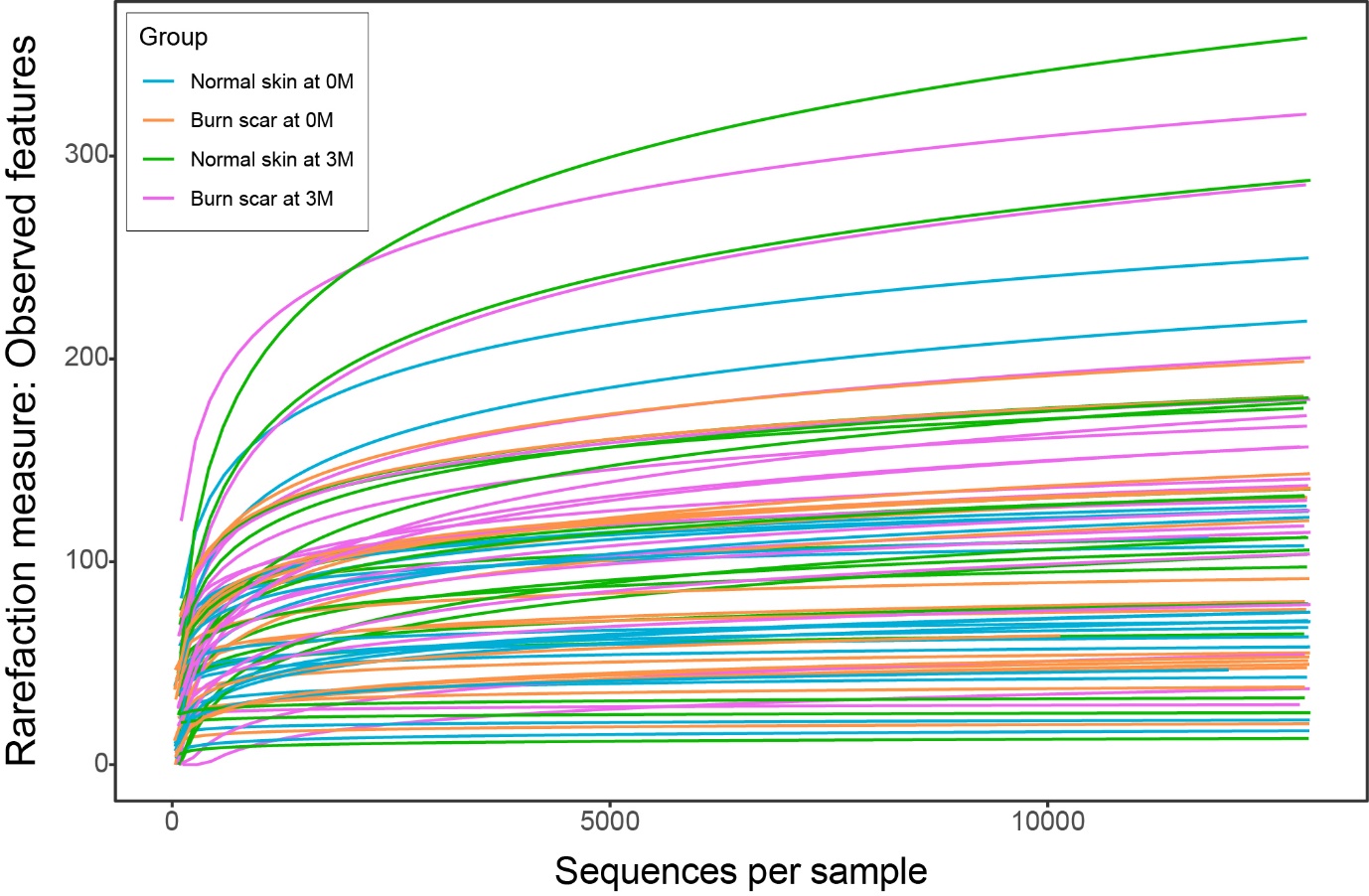
**

**Supplementary figure 1.** Rarefaction curve of all skin sample.


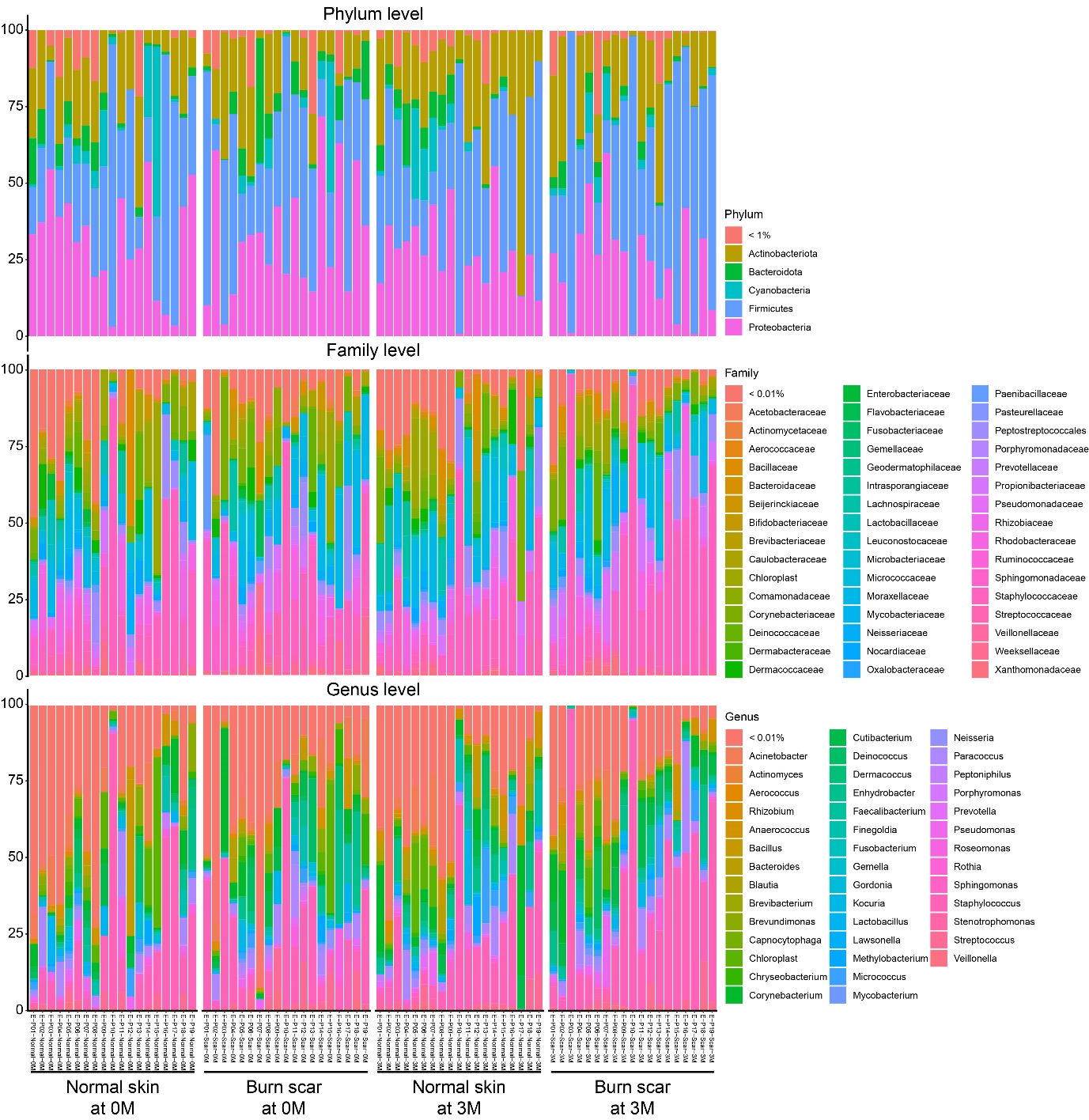


**Supplementary figure 2.** Relative microbial composition of the treated and untreated samples at the baseline (0M) and after three months (3M) of extracorporeal shock wave therapy in individual patients at the phylum, family, and genus level
